# Motivations for crystal methamphetamine-opioid co-injection/co-use amongst community-recruited people who inject drugs: a qualitative study

**DOI:** 10.1186/s12954-020-00360-9

**Published:** 2020-02-27

**Authors:** Anna Palmer, Nick Scott, Paul Dietze, Peter Higgs

**Affiliations:** 1 Behaviours and Health Risks Program, Burnet Institute, 85 Commercial Road, Melbourne, VIC 3004 Australia; 2 Department of Epidemiology and Preventive Medicine, Monash University, 553 St Kilda Road, Melbourne, VIC 3004 Australia; 3 Department of Public Health, La Trobe University, Plenty Rd & Kingsbury Dr, Bundoora, VIC 3086 Australia

**Keywords:** Methamphetamine, Opioids, Injecting drug use, Opioid substitution therapy, Harm reduction, Polydrug use, Co-injection, Qualitative

## Abstract

**Aims:**

We report on motivations for crystal methamphetamine-opioid co-use/co-injection through narratives of people who inject drugs during a period of increased crystal methamphetamine use reporting in Australia.

**Methods:**

Fourteen in-depth interviews were undertaken with selected participants (12 male, 2 female) from the Melbourne Injecting Drug User Cohort Study, including those in and out of opioid substitution therapy (OST).

**Results:**

The main motivations for co-use reported by participants were as follows: (1) that heroin could be used to reduce the negative side effects of heavy crystal methamphetamine use, particularly during the ‘comedown’ phase; (2) that small quantities of crystal methamphetamine used with heroin could prolong the intoxication effect of heroin, and hence the time before opioid withdrawal; (3) that co-injection of crystal methamphetamine and heroin produced a more desirable intoxication effect than using either substance on its own and; (4) that crystal methamphetamine provided a substitute ‘high’ for heroin after commencing OST treatment.

**Conclusions:**

Co-use of methamphetamine and opioids has been used by people who inject drugs to facilitate intoxication, sometimes as the result of ineffective opioid substitution therapy (OST) treatment and perceived lack of pleasure after stabilisation on OST treatment.

## Introduction

Co-use of opioids and stimulants has been a practice widely documented in studies of people who inject drugs (PWID) [1]. Approximately 13% of PWID participants in recent Australian drug monitoring surveys reported using a combination of opioids and stimulants on the day preceding their survey [2]. Whilst some studies suggest that opioid and stimulant co-use may be positively associated with risk of overdose [3, 4] and poorer treatment outcomes for those on opioid substitution therapy (OST) [5–8], a current lack of co-use/co-injection data means that the epidemiology and impacts of the practice remain largely understudied.

Over the past two decades, Australian methamphetamine markets have been in flux, with parts of the country witnessing the peak of a second methamphetamine ‘epidemic’ in 2014 [9, 10]. This was in part sparked by a shift in the predominant form of methamphetamine used (from ‘speed’ to crystal methamphetamine—a typically stronger form) [11] and increased purity of all methamphetamine forms [12] coinciding with increased reporting of crystal methamphetamine-related harms amongst stimulant users and PWID [13]. However, the impact of these drug market changes on opioid-stimulant co-use are unknown and there has been little research on the motivations for co-using/co-injecting methamphetamine with opioids in Australia or internationally.

Motivations for opioid-stimulant co-use have previously been explored in the context of the ‘crack (cocaine) epidemic’ of the late 1980s and early 1990s in the USA. These studies suggested that individuals were using a combination of heroin and cocaine to modulate the severity of opiate withdrawal [14–16], or to help deal with the over-excitability associated with ‘binge’ cocaine use [14, 17]. Other studies suggested that co-administering low doses of heroin and cocaine was more ‘reinforcing’ than using either substance alone [18] and that people who were prescribed methadone used cocaine as a way to replace the intoxication they formerly gained from heroin [15, 19]. These studies also revealed the use of heroin and cocaine in the same injection—referred to as ‘speedballing’ [1, 14]. Overall, these studies suggest that motivations for co-using stimulants and opioids are complex, multifactorial and context dependent, making them difficult to report on.

One way to examine risk behaviours (such as heavy drug use and risky sexual behaviour) is by considering them within the frame of ‘situated rationality’ where potentially harmful behaviours, appearing irrational to an outsider, can be seen as rational from an individual’s perspective [20]. Linked to this framing are physical, social, economic and political factors—often termed the ‘risk environment’—which comes to shape risk behaviour [21]. This framework has previously been used to explore social and contextual factors that limit the effectiveness of heroin overdose education, arguing that current prevention strategies (such as encouraging users to avoid the concurrent use of other depressant drugs, to sample their heroin strength prior to using and to avoid using alone) can be ineffective because they disregard the social, cultural and economic realities of street-based PWID [22, 23]. By using the ‘risk environment’ approach, Rhodes [21] argues that a more comprehensive approach to harm reduction is created, raising the importance of ‘non-drug’ and ‘non-health’ interventions and increasing understanding of how drug-related harms intersect health and vulnerability. Such an understanding has led to the importance of considering pleasure in accounts of drug consumption (alongside harm reduction), in order to generate more successful drug policy conversations [24]. Although we do not explore the sociological and anthropological consequences of this (or other frameworks) in-depth, approaches like these can provide a powerful framing for exploring opioid-stimulant co-use, and avoid rigid, de-contextualised understandings which have proven unconstructive in the past.

In this study, we explore motivations for crystal methamphetamine-opioid co-use/co-injection amongst PWID during a period of heightened crystal methamphetamine use reporting, guided by the ‘risk environment’ framework.

## Methods

### Design

We adopted a qualitative approach in this study, drawing upon participant narratives of methamphetamine-heroin co-use, methamphetamine-heroin co-injection and concomitant use of methamphetamine with OST.

### Participants

Participants were recruited from the Melbourne Injecting Drug User Cohort Study (MIX)—a longitudinal cohort study of PWID aiming to examine trajectories of injecting drug use and associated outcomes. MIX initiated recruitment in 2008 and is currently ongoing; the details of recruitment, data collection and follow-up procedures are published elsewhere [25]. Between May 2016 and August 2016, participants from MIX were approached and asked to complete an in-depth interview. Participants were purposively sampled based on whether they reported engaging in a period of heavy crystal methamphetamine use since recruitment (defined as a period where crystal methamphetamine was the drug they were using most).

### Data collection

In-depth interviews were guided by a short list of topics—crystal methamphetamine/opioid patterns of use, crystal methamphetamine harms, crystal methamphetamine-opioid co-injection and crystal methamphetamine use with OST. Interviews took place at a variety of locations (including in needle and syringe programs, cafes, parks and within a dedicated mobile research van) at the discretion of the participant ensuring adequate privacy was obtained. A.P. conducted the interviews (overseen by P.H.). Interviews lasted approximately 1 h and were audio-recorded, then later transcribed verbatim.

### Analysis

We used a narrative analysis, guided by the ‘risk environment’ framework as suggested by Rhodes [21]. This meant that we used PWID-specific contextual factors (including factors relating to the drug market environment, drug user social networks and drug treatment) to guide the interview topics and analysis. Coding began with deductive codes (informed by the interview guide); however, inductive codes were added to the analysis as they arose. After initial open coding of interview transcripts, codes were organised into the themes/topics emerging across interviews using qualitative analysis computer software. A.P. initially coded the interviews and shared her work with P.H. who read the interviews and added input on the initial coding analysis. After discussion amongst A.P. and P.H., a draft report was written up and shared with the remaining authors (who did not themselves read the full interview transcripts but discussed the analysis in the report). Participant identities were concealed (only gender and age reported), and identifying names, places and events were not disclosed in the analysis.

## Results

### The participants

Sixteen potential participants were approached and invited to participate; 2 declined citing privacy concerns. Of the 14 participants who were recruited, 2 were female. The average age at the time of interview was 35 (range 29–39). All participants reported themselves to be opioid dependent (having initiated their injecting career during their teens) but also reported engaging in polydrug use. Ten participants were prescribed OST at the time of the interview.

### Opioids reduce negative side effects of crystal methamphetamine

All participants reported that they were engaging in heavy periods of crystal methamphetamine use, with most reporting that this coincided with a rise in crystal methamphetamine availability in their local area. During periods of heavy use participants reported injecting crystal methamphetamine multiple times to obtain an intoxicated state, which lasted anywhere between 24 h and 2 weeks. Participants found it difficult to articulate why they were using such large quantities of crystal methamphetamine, often implying that they were unable to control their own usage. One participant stated that he was unaware of how heavy crystal methamphetamine use was affecting him for several months:

It went on for about six months, I was using every day. I didn’t know how fucked up I got until I got locked up and went to jail. I slept for ten days straight. I didn’t even get up to eat. (Participant 1, male, 33 years)

This participant explained that periods of heavy use typically stemmed from successive nights of partying, selling drugs and using. Other participants described similar experiences, explaining that they were using large quantities of crystal methamphetamine (a substance they considered ‘strong’) leading them to feel out of control both physically and mentally:

… it kept me up for such a long time. And this was just smoking it. I would be up for days and I would be using heroin to try and put me to sleep but nothing would work. I had no control. (Participant 2, male, 39 years)

Despite the lack of control participants felt when intoxicated on crystal methamphetamine, all participants reported continuing heavy use. Furthermore, participants also reported negative experiences during the ‘comedown’ phase of crystal methamphetamine intoxication. Sweats, shakes, dehydration, fainting and drug-induced psychotic episodes were reported as common symptoms during this phase, and it was evident that participants found these symptoms disturbing:

… when you are coming down you can feel it, you sweat it out of your skin and it stinks. It is revolting. (Participant 3, male, 36 years)

To ‘treat’ these effects, participants reported using a range of depressant substances. Whilst the most common substance was heroin, participants also reported using cannabis, benzodiazepines and mood stabilisers. These substances reportedly helped participants regain physical and mental control and often helped participants sleep after extended periods of crystal methamphetamine intoxication:

…when I’m coming down off it, I pretty much just go and get heroin, then I feel a bit better. But yeah, I can’t stand coming off it. It’s bad feelings. (Participant 4, male, 38 years)

I like the feeling of it [heroin], like just the way that it helps you sleep. And like, when I’m coming off the ice, just to get back down… coming off one hundred miles an hour (Participant 5, male, 35 years)

This pattern of co-use (using heroin during the ‘comedown’ phase of a crystal methamphetamine ‘high’) was commonly reported amongst participants, with many participants opting to do this if they were financially able; some participants reported that they often did not have enough money to purchase heroin to ‘comedown’.

### Crystal methamphetamine prolongs heroin intoxication and forestalls opioid withdrawal

Participants reported that avoiding/alleviating the symptoms of opioid withdrawal was a primary concern for them. This included participants enrolled in OST programs, as many reported missing doses or that their prescribed treatment was not high enough to ‘hold them’. Participants reported that one injection of heroin would typically only satisfy their withdrawal symptoms temporarily and that after this time they were left ‘hanging out’ and in need of another injection:

Interviewer: How long would the heroin [injection of heroin] usually last for? Participant: I’d have to have a shot [injection], every 4 hours just to… yeah [stay intoxicated]. (Participant 5, male, 35 years)

During interviews, the topic of ‘cocktailing’ crystal methamphetamine and heroin (the use of crystal methamphetamine and heroin in the same injection) was frequently discussed by participants. A few participants reported that by combining heroin and crystal methamphetamine in the same injection, they could extend the effects of the heroin and therefore forestall the onset of opioid withdrawal symptoms. Participants loosely explained that this was due to the combination of depressant-stimulant effects, where they perceived that the stimulant drug prolonged the effect of the depressant:

For heroin on its own I could have a taste of heroin, then five minutes later I would want another one. Whereas if I had a cocktail, I’ll have one and I’ll be alright for about eight hours. It doesn’t come to mind and I don’t crave it, so I use less of it. (Participant 6, male, 31 years)

For these participants, the effect of combining the two substances meant that they were able to use less heroin to avoid opioid withdrawal; an outcome which was not only perceived to be physically beneficial but also cost-effective.

### The combination of crystal methamphetamine and heroin ‘feels better’

Upon further discussion of crystal methamphetamine-heroin ‘cocktailing’, a few participants reported that the combination of crystal methamphetamine and heroin in the same injection ‘felt better’ than using either substance alone. Participants reported that the crystal methamphetamine could ‘boost’ the effect of the heroin, leading to a more desirable high. It was evident that these participants enjoyed this intoxication, and became accustomed to practising this regularly:

In this combination, it actually boosts the heroin and makes the heroin sensation stronger. It brings on the aroma of the heroin but you are also in this state where you want to be tinkering and doing things, or where you can drift off… (Participant 2, male, 39 years)

In further describing this practice, participants reported that it was important to use the correct quantities of crystal methamphetamine and heroin. Because the stimulant effects of crystal methamphetamine were strong, participants reported using only small quantities of crystal methamphetamine within the mix (the percentage reported between 5 and 20%) with the remainder of the mix being heroin:

Because ice is so strong, if you put one part heroin and one part ice, the ice will override the heroin so you basically waste your heroin. You won’t feel heroin. Even if you put half that, half ice to your heroin it is still too strong. I work at about a 5% to 10% ratio. (Participant 2, male, 39 years)

Although not all participants reported engaging in ‘cocktailing’ (some preferred the effects of each substance on its own), all participants were familiar with the practice, and many had tried it at least once.

### Crystal methamphetamine replaces a heroin ‘high’ when on OST

Participants who were in an OST program at the time of the interview reported using crystal methamphetamine on days they were taking prescribed doses, and on days they were not. When regularly adhering to their OST regimen, many participants reported that the treatment satiated their physical opioid-dependency symptoms but not their psychological desire for intoxication. Thus, these participants reported seeking a ‘high’ from secondary substances, such as crystal methamphetamine, which would not be affected by their OST treatment. For a number of participants, using crystal methamphetamine on top of their OST treatment became a way of substituting a heroin ‘high’, and thus satisfying their psychological need for intoxication:

Yeah, it’s [crystal methamphetamine] a bit of a substitute, a big substitute actually. It just sort of satisfies me, much the same way that heroin did. (Participant 5, male, 35 years)

A common theme amongst participants was the perceived need to feel ‘high’, as participants reported they did not feel comfortable when they were not. Participants referred to the state of no intoxication as being ‘straight’ and explained that this state often made them feel anxious or depressed. Furthermore, participants on OST reported that they often felt ‘straight’ when regularly adhering to their OST treatment regimen and that even though they felt their physical opioid-dependency symptoms had been alleviated, the desire for intoxication remained. As one participant explained:

You just sort of need something to, you know, use, as opposed to being straight. It’s not enough. That’s the problem. It just keeps your mind off everything. You think too much when you are straight. When you use you don’t think as much, you know what I mean? (Participant 7, male, 35 years)

## Discussion

Our study reveals motivations for co-using/co-injecting crystal methamphetamine and heroin amongst opioid-dependent PWID. These findings suggest that co-use is a complex behaviour, which is heavily influenced by an individuals’ drug use history and their physical/psychological dependency on opioids. In many cases, co-use was reported as a way of regaining control during heavy periods of crystal methamphetamine use, reducing the negative side effects of opioid withdrawal and satisfying the perceived psychological need to be intoxicated. However, co-use was also found to simply reflect an increased availability of methamphetamine in participants’ local drug market. These findings suggest that achieving and maintaining intoxication was a primary concern for participants that co-using methamphetamine and opioids facilitated a desired state of intoxication and that this was facilitated by local drug market contexts.

Although stimulant-opioid co-use is typically viewed by the public as a ‘risky’/‘harmful’ practice, our study has shown that such behaviour can be perceived as rational from the individual’s perspective. It became clear that many participants felt the need to be intoxicated (even after their physical cravings for opioids were satisfied by OST) and that co-use was a method of facilitating this, either by allowing them to achieve intoxication whilst on OST (and hence alleviate symptoms of worry or anxiety) or regain control during heavy periods of methamphetamine use. Furthermore, participants reported using a combination of methamphetamine and heroin, either as a way of enhancing intoxication, or extending the intoxication period to avoid opioid withdrawal—a universal concern amongst participants. Combined with the reported increase in crystal methamphetamine availability at the time, these findings emphasise the key role of external features of the ‘risk environment’ in influencing co-use of crystal methamphetamine and opioids, and the importance of ‘drugged pleasures’ in participants’ lives. As argued by Dennis and Farrugia [24], such considerations (as we have presented here) have often been absent in drug research and neglected from current drug policy debates.

There are a number of key implications highlighted by our study findings. Firstly, it was clear that OST treatments did not meet the needs of many participants in the study, leading them to compensate through primary/secondary substance use. Thus, our study not only emphasises that dosing regimens should continue to be carefully assessed but also managed in consultation with the patients themselves. We also note that other elements of OST such as the pharmacological characteristics of specific drugs may not be meeting the needs of individuals who are opioid dependent. Specifically, our findings reveal that the pharmacological characteristics of buprenorphine when used as OST can cause individuals to feel deprived of heroin-related pleasure leading them to use secondary substances such as methamphetamine. Whilst some drug policy debate focusses strongly on abstinence-based approaches, findings such as these suggest that individuals could be better served by alternative approaches, which focus on overall health and well-being (as opposed to complete abstinence). Such approaches would consider the concept of pleasure as a part of general health and well-being, in order to address the needs of individuals who are opioid dependent. Secondly, whilst our findings reveal that some PWID engage in methamphetamine-opioid co-injection, we are unable to report on any specific harms associated with the practice, and to our knowledge, the prevalence and/or harms associated with this practice in Australia are largely unknown. Studies from other regions suggest that prevalence of co-injection could be as high as 39.9% amongst PWID [4]; however, further investigation is required to determine the local prevalence of the practice once data become available.

There are a number of limitations in this study. Firstly, we recruited participants as a convenience sample, meaning that only participants who were contactable by researchers were invited to participate (typically those who were due for annual follow-up for the MIX study). Secondly, we relied heavily on self-reporting of behaviour by participants. This means that our data may be subject to inaccuracies due to mis-remembering events, and under-reporting sensitive experiences or behaviour which participants may have felt stigmatised by reporting. Efforts were made to ensure that participants felt comfortable sharing their experiences and that they would not be stigmatised by participating in this study. Thirdly, we had insufficient data to assess gender differences in participant narratives. Whilst 14% of our sample (2/14) was female, larger scale studies of PWID (such as MIX) suggest that approximately one third of PWID are female. As such, the conclusions presented in this paper may not sufficiently reflect the views and opinions of female PWID. Future studies should seek to recruit more females to assess any such differences.

## Conclusions

Our study reveals that the co-use of methamphetamine and opioids is shaped by motivations ranging from the simple availability of methamphetamine through to personal perceptions of the intoxicating effects of the combination. Specifically, our findings indicate that co-use of stimulants and opioids can present as an effect or symptom of more fundamental issues faced by opioid-dependent individuals, such as ineffective opioid dependency treatments and a perceived lack of pleasure from their lives. Approaches to harm reduction for opioid-stimulant co-use should reflect the context of the behaviour and address the specific needs of opioid-dependent individuals.

## Data Availability

The datasets used and/or analysed during this study are available from the corresponding author on reasonable request.
